# Methodological considerations on real-world evidence studies of monoclonal antibodies against the CGRP-pathway for migraine: a systematic review

**DOI:** 10.1186/s10194-023-01611-3

**Published:** 2023-06-22

**Authors:** Nicolas Vandenbussche, Karolina Pisarek, Koen Paemeleire

**Affiliations:** 1 Department of Neurology, Ghent University Hospital, Corneel Heymanslaan 10, B-9000 Ghent, Belgium; 2 Faculty of Medicine and Health Sciences, Ghent University, Corneel Heymanslaan 10, B-9000 Ghent, Belgium

**Keywords:** Methodology, Migraine, CGRP, Real-world evidence, Clinical trials

## Abstract

**Background:**

Real-world data are accumulating on the effectiveness, tolerability and safety of anti-calcitonin gene-related peptide pathway monoclonal antibodies for the preventive treatment of migraine. We performed a systematic review of the methodology of prospective, observational, clinic-based real-world evidence studies with these drugs in both episodic and chronic migraine.

**Methods:**

The objectives were to evaluate the definitions and reported outcomes used, and to perform a risk of bias assessment for each of the different studies. PubMed and EMBASE were systematically queried for relevant scientific articles. Study quality assessment of the included studies was conducted using the “National Heart, Lung and Blood Institute (NHLBI) Study Quality Assessment Tool for Before-After (Pre-Post) Studies with No Control Group”.

**Results:**

Forty-six studies fitted the criteria for the systematic review and were included in the analysis. Ten studies (21.7%) defined a migraine day for the study, while only 5 studies defined a headache day for the study (10.9%). The most common primary endpoint/objective of the studies was change in monthly migraine days (*n* = 16, 34.8%), followed by responder rate (*n* = 15, 32.6%) and change in monthly headache days (*n* = 5, 10.9%). Eight studies (17.4%) did not define the primary endpoint/objective. Thirty-three studies were graded as “good” quality and 13 studies were graded as “fair”.

**Conclusion:**

Our analysis shows rather significant heterogeneity and/or lack of predefined primary outcomes/objectives, definitions of outcomes measures and the use of longitudinal monitoring (e.g. headache diaries). Standardization of terminology, definitions and protocol procedures for real-world evidence studies of preventive treatments for migraine are recommended.

**Trial registration:**

This study was registered with PROSPERO with ID CRD42022369366.

**Supplementary Information:**

The online version contains supplementary material available at 10.1186/s10194-023-01611-3.

## Introduction

The arrival of anti-calcitonin gene-related peptide (CGRP) pathway monoclonal antibodies for the treatment of migraine has significantly impacted the clinical field of headache medicine in recent years. Several monoclonal antibodies have been tested in large randomized clinical trials with positive results in terms of efficacy, tolerability and safety in both episodic (EM) and chronic migraine (CM) [1–23]. Since 2018, four monoclonal antibodies have been approved by regulatory agencies and are commercially available: eptinezumab, erenumab, fremanezumab and galcanezumab.

The settings of a clinical trial and the profile of its participants may however not fully reflect everyday practice. Real-world data (RWD) from routine clinical care allow to assess effectiveness (as opposed to efficacy) of new treatments, to compare new treatments to the standard of care (useful in guideline development), and provide postmarketing safety information. According to the FDA, RWD are “the data relating to patient health status and/or the delivery of health care routinely collected from a variety of sources” [24]**.** Analysis of RWD can generate Real-World Evidence (RWE), “the clinical evidence regarding the usage and potential benefits or risks of a medical product derived from analysis of RWD” [24].

Prospective and non-randomized observational studies reflect everyday practice best but are of lower quality compared to randomized controlled trials in the hierarchy of evidence-based medicine [25]. The former studies however may help in understanding real-world experiences from clinicians treating patients with new therapeutics. RWE studies can also be advantageous in collecting data from patient groups which may be excluded from randomized controlled trials, such as patients in higher age groups, patients with high numbers of prior preventive treatment failures or patients with certain comorbid conditions. Quality control of those studies should be equally rigorous. Methodological considerations made during study design and the reporting of methodology within a scientific article are profoundly important aspects of evidence-based medical research. When the methodological quality of the study is high, the RWE generated can be regarded as complementary to data from randomized controlled trials [26]. RWD of the highest quality may be used for decision making processes by regulatory medicine agencies [24, 27, 28].

The International Headache Society (IHS) has created guidelines for the development and conductance of clinical trials, but recommendations or guidelines for the collection of RWD from prospective observational studies are currently not available. Standardization of definitions, baseline characteristics and outcome measures is needed to understand treatment effects and to compare different studies [29–31].

In this systematic review, we study the methodology of prospective, observational, clinic-based studies investigating effectiveness, tolerability and safety of anti-CGRP pathway monoclonal antibodies for the treatment of migraine. The primary objectives of this systematic review are 1) to summarise the used definitions within these studies, 2) to investigate the reported outcomes used and 3) to perform a risk of bias assessment for each of the different studies.

## Methods

This systematic review was prospectively registered with PROSPERO (CRD42022369366) and adhered to the Preferred Reporting Items for Systematic Reviews and Meta-Analyses (PRISMA) guidelines [32–35]. A systematic search within the databases of MEDLINE (PubMed interface) and Embase was developed and performed by authors NV and KPi (Table 1). Query results were filtered between May 1^st^ 2018 (i.e. following the approval of erenumab by the FDA, the first market authorization for an anti-CGRP pathway monoclonal antibody globally) and September 30^th^ 2022 (pre-determined end date). The search queries can be found below. Articles were screened by reading the titles, abstracts and keywords and if needed the full text.

**Table 1:** Systematic search for the databases of MEDLINE (PubMed interface) and Embase

PUBMED
((antibod*[Title/Abstract]) AND ((cgrp[Title/Abstract]) OR (calcitonin gene-related peptide[Title/Abstract]))) OR ((erenumab[Title/Abstract]) OR (aimovig[Title/Abstract]) OR (AMG-334[Title/Abstract]) OR (AMG334[Title/Abstract]) OR (erenumab-aooe[Title/Abstract]) OR (galcanezumab[Title/Abstract]) OR (emgality[Title/Abstract]) OR (LY2951742[Title/Abstract]) OR (LY-2951742[Title/Abstract]) OR (galcanezumab-gnlm[Title/Abstract]) OR (eptinezumab[Title/Abstract]) OR (vyepti[Title/Abstract]) OR (ALD403[Title/Abstract]) OR (ALD-403[Title/Abstract]) OR (eptinezumab-jjmr[Title/Abstract]) OR (fremanezumab[Title/Abstract]) OR (ajovy[Title/Abstract]) OR (TEV-48125[Title/Abstract]) OR (TEV48125[Title/Abstract]) OR (fremanezumab-vfrm[Title/Abstract]))
**EMBASE**
'erenumab':ti,ab OR 'aimovig':ti,ab OR 'amg-334':ti,ab OR 'amg334':ti,ab OR 'erenumab-aooe':ti,ab OR 'galcanezumab':ti,ab OR 'emgality':ti,ab OR 'ly2951742':ti,ab OR 'ly 2,951,742':ti,ab OR 'galcanezumab-gnlm':ti,ab OR 'eptinezumab':ti,ab OR 'vyepti':ti,ab OR 'ald403':ti,ab OR 'ald-403':ti,ab OR 'eptinezumab-jjmr':ti,ab OR 'fremanezumab':ti,ab OR 'ajovy':ti,ab OR 'tev-48125':ti,ab OR 'tev48125':ti,ab OR 'fremanezumab-vfrm':ti,ab OR (('cgrp':ti,ab OR 'calcitonin gene-related peptide':ti,ab) AND 'antibod*':ti,ab) AND (2018:py OR 2019:py OR 2020:py OR 2021:py OR 2022:py)

### Study selection, inclusion and exclusion criteria

Following the retrieval of the query results for all databases, two authors (NV and KPi) independently screened the individual abstracts for eligibility. The full texts of records deemed eligible were retrieved after which two authors (NV and KPi) independently read and evaluated the manuscripts for inclusion. When confronted with discordance, a decision based on consensus after retrieval of the full text was done by both reviewing authors (NV and KPi).

We included research articles providing prospective, clinic-based, observational data on adult human subjects treated with anti-CGRP pathway monoclonal antibodies and with primary endpoints or outcomes of effectiveness, tolerability and safety of these drugs in clinical practice. Studies could only be initiated by clinical researchers, not by pharmaceutical companies. The research article had to declare the prospective design of the study. Studies reporting on participants with EM and/or CM, with or without medication-overuse headache (MOH), were eligible. Exclusion criteria were: articles with an experimental primary focus not involving effectiveness, tolerability or safety of the drug; retrospective analyses; pharmaco-economic database studies; systematic reviews and meta-analyses. There were no geographical restrictions but papers needed to be written in English.

Research articles fitting the criteria formulated above were independently assessed in the systematic review on their own methodology, predefined outcomes and definitions and reported outcomes. Therefore, multiple manuscripts which were part of a larger research effort by the same study group remained analysed separately for the following reasons: 1) fully equivalent methodological approaches in terms of outcomes or definitions across manuscripts could not be assumed a priori to the analysis, 2) the focus of this review was on the reporting standards and the methodological considerations of the manuscripts rather than the actual collected study results.

### Data extraction

A structured digital form was established for data collection. Data points on the following characteristics were collected: general study characteristics, treatment regimens, headache-related definitions utilized within the study, baseline characteristics of participants, headache characteristics (medical history, symptomatology, medication usage), headache diary usage, primary and secondary endpoints and objectives, usage of validated questionnaires, collection of tolerability and safety aspects (adverse events, serious adverse events, discontinuation rates and aspects and pregnancies during registration). Outcomes analysed were based on the IHS guidelines for controlled trials of preventive treatment of CM in adults [31]**.** Possible outcomes for a data point were “present”, “not present” or “no information”. Unless the authors specifically stated that a data point was not available or not reported in the study manuscript, all information that was missing was documented as “no information”.

### Study quality assessment

Study quality assessment (i.e. good, fair or poor) of the included studies was conducted using the “National Heart, Lung and Blood Institute (NHLBI) Study Quality Assessment Tool for Before-After (Pre-Post) Studies with No Control Group” [36, 37]. Two authors (NV and KPi) assessed the quality of each manuscript independently by applying this tool. When confronted with disconcordance, a decision based on consensus was made by both authors (NV and KPi).

## Results

### Included articles

**Fig. 1:**
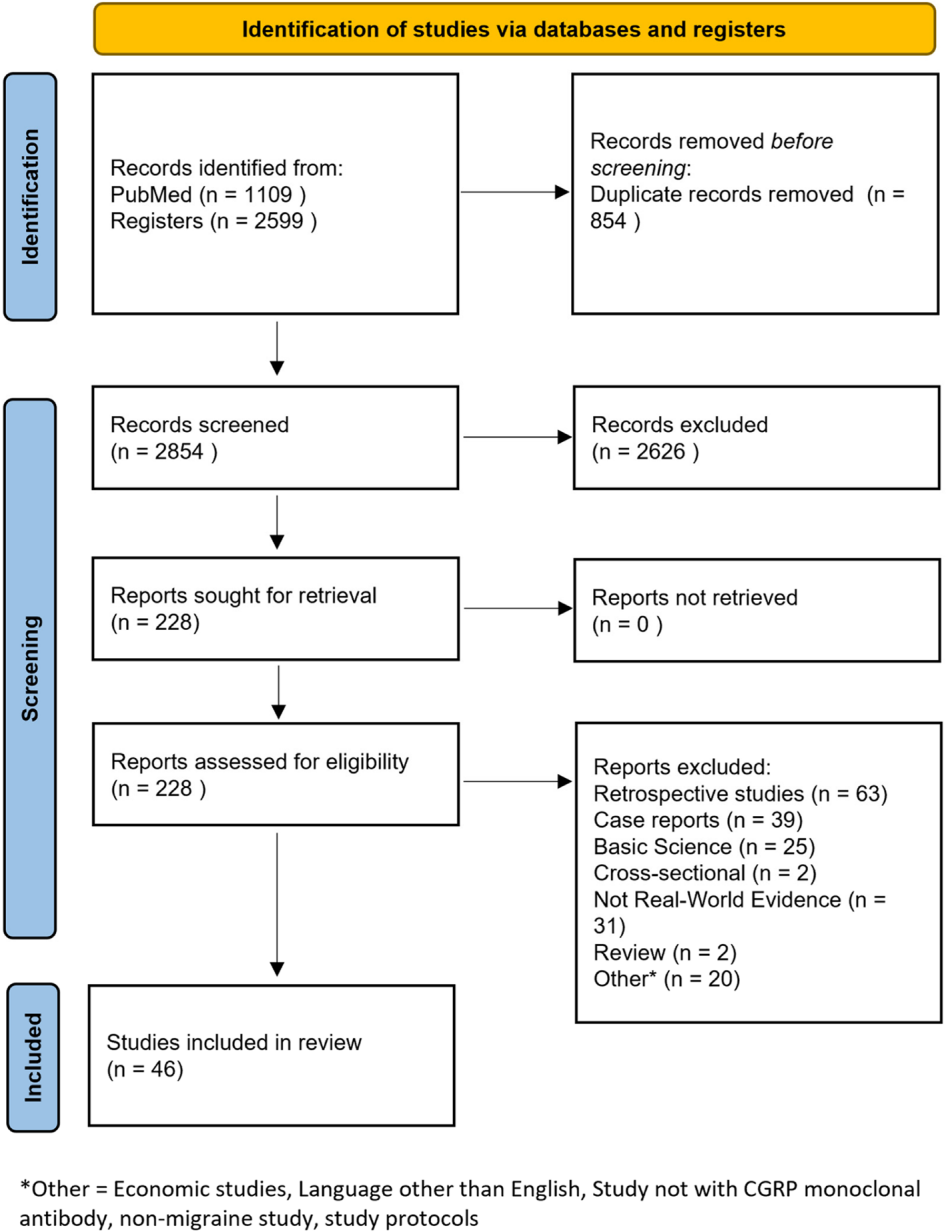
PRISMA 2020 flow diagram for systematic reviews

Both queries resulted in a total of 3788 articles (PubMed = 1109 results, EMBASE = 2599 results). After removal of duplications and screening of records, 228 records were assessed in more depth for eligibility. Finally, 46 studies fitted the criteria for the systematic review and were analysed [38–83]. The flow diagram can be found in Fig. 1. Included studies can be found in Table 2.

**Table 2:** Included studies (*N* = 46)

First Author	N	Diagnosis	Drugs	Country	Setting	Start date	End date
**Alpuente 2021 **[38]	263	EM and CM	Erenumab, Galcanezumab	Spain	Monocentric	February 2019	February 2021
**Alpuente 2021 **[39]	155	CM	Erenumab, Galcanezumab	Spain	Monocentric	NA	NA
**Altamura 2022 **[40]	155	EM and CM	Galcanezumab	Italy	Multicentric	September 2019	December 2021
**Barbanti 2019 **[42]	78	EM and CM	Erenumab	Italy	Monocentric	20 December 2018	7 March 2019
**Barbanti 2020 **[43]	372	EM and CM	Erenumab	Italy	Multicentric	December 2018	September 2019
**Barbanti 2021 **[41]	242	EM and CM	Erenumab	Italy	Multicentric	December 2018	July 2020
**Barbanti 2022 **[44]	53	EM and CM	Fremanezumab	Italy	Multicentric	July 2020	November 2020
**Becker 2022 **[45]	95	EM and CM	Erenumab	Canada	Multicentric	April 2019	April 2020
**Belvis 2021 **[46]	210	EM and CM	Erenumab	Spain	Multicentric	February 2019	June 2020
**Caronna 2021 **[47]	139	CM	Erenumab	Spain	Monocentric	February 2019	April 2021
**Cetta 2022 **[48]	30	EM and CM	Erenumab	Italy	Monocentric	February 2019	January 2022
**Cheng 2020 **[49]	170	CM	Erenumab	Australia	Multicentric	October 2018	April 2020
**Cullum 2022 **[50]	300	CM	Erenumab	Denmark	Monocentric	January 2019	February 2020
**Curone 2020 **[51]	27	CM	Erenumab	Italy	Monocentric	NA	NA
**Curone 2022 **[52]	303	CM	Erenumab, Galcanezumab, Fremanezumab	Italy	Monocentric	October 2020	September 2021
**De Icco 2022 **[53]	77	CM	Erenumab	Italy	Monocentric	December 2018	January 2020
**De Matteis 2021 **[54]	32	EM and CM	Erenumab	Italy	Multicentric	December 2019	October 2020
**de Vriesch Lentsch 2021 **[55]	100	EM and CM	Erenumab	The Netherlands	Monocentric	January 2019	March 2020
**Gonzalez-Martinez 2022 **[56]	712	EM and CM	Erenumab, Galcanezumab, Fremanezumab	Spain	Multicentric	November 2018	August 2021
**Guerzoni 2022 **[57]	185	CM	Erenumab	Italy	Multicentric	NA	NA
**Iannone 2022 **[59]	44	CM	Erenumab, Galcanezumab	Italy	Monocentric	December 2019	June 2020
**Iannone 2022 **[58]	203	CM	Erenumab, Galcanezumab, Fremanezumab	Italy	Monocentric	December 2019	April 2021
**Krymchantowski 2022 **[60]	112	EM and CM	Erenumab, Galcanezumab, Fremanezumab	Brazil	Multicentric	February 2020	March 2021
**Kwon 2022 **[61]	92	EM and CM	Galcanezumab	South Korea	Monocentric	June 2020	April 2021
**Lambru 2020 **[62]	164	CM	Erenumab	United Kingdom	Monocentric	October 2018	September 2019
**Lowe 2022 **[63]	103	CM	Erenumab	United Kingdom	Monocentric	March 2020	December 2020
**Mahovic 2022 **[64]	57	CM	Erenumab	Croatia	Monocentric	March 2019	November 2019
**Matteo 2020 **[65]	159	EM and CM	Erenumab	Italy	Monocentric	May 2019	April 2020
**Pensato 2020 **[68]	39	CM	Erenumab	Italy	Monocentric	May 2019	May 2020
**Pensato 2021 **[66]	149	CM	Erenumab	Italy	Multicentric	May 2019	May 2020
**Pensato 2022 **[67]	111	CM	Erenumab, Galcanezumab	Italy	Multicentric	April 2019	November 2020
**Raffaelli 2022 **[69]	39	EM and CM	Erenumab, Galcanezumab, Fremanezumab	Germany	Monocentric	NA	NA
**Raffaelli 2022 **[70]	62	EM and CM	Erenumab, Galcanezumab, Fremanezumab	Germany	Monocentric	January 2020	November 2020
**Ranieri 2020 **[71]	30	EM and CM	Erenumab	Italy	Monocentric	April 2019	May 2020
**Russo 2020 **[72]	70	CM	Erenumab	Italy	Monocentric	February 2019	July 2019
**Saeed 2022 **[73]	90	EM and CM	Erenumab	NA	Monocentric	NA	NA
**Silvestro 2022 **[74]	43	EM and CM	Galcanezumab	Italy	Multicentric	January 2021	June 2021
**Terhart 2021 **[75]	61	EM and CM	Erenumab, Galcanezumab, Fremanezumab	Germany	Monocentric	January 2020	December 2020
**Torres-Ferrus 2021 **[76]	155	EM and CM	Erenumab	Spain	Monocentric	February 2019	October 2020
**Tziakouri 2021 **[77]	16	CM	Erenumab	Cyprus	Monocentric	NA	NA
**Vernieri 2020 **[78]	81	EM and CM	Galcanezumab	Italy	Multicentric	NA	July 2020
**Vernieri 2021 **[79]	156	CM	Galcanezumab	Italy	Multicentric	November 2019	January 2021
**Vernieri 2021 **[80]	163	EM and CM	Galcanezumab	Italy	Multicentric	November 2019	January 2021
**Vernieri 2021 **[82]	154	EM and CM	Erenumab, Galcanezumab	Italy	Multicentric	November 2019	July 2021
**Vernieri 2022 **[81]	191	EM and CM	Galcanezumab	Italy	Multicentric	September 2019	November 2021
**Zecca 2022 **[83]	110	EM and CM	Erenumab	Switzerland	Multicentric	December 2019	September 2020

Legend: *CM* chronic migraine, *EM* episodic migraine, *NA* not available, *N* number of participants

### *General studycharacteristics*

All 46 studies included participants of male or female sex [38–83]. Twenty-seven studies included participants with either EM or CM [38, 40–46, 48, 54–56, 60, 61, 65, 69–71, 73–76, 78, 80–83]. Nineteen studies only looked at participants with CM [39, 47, 49–53, 57–59, 62–64, 66–68, 72, 77, 79]. Forty studies (87%) reported the use of the ICHD-3 criteria for the diagnosis [38, 40, 43–62, 64–72, 74–77, 79–84].

Studies had a median number of 111 participants (interquartile range 61 to 164 participants). Nineteen studies (41.3%) formulated exclusion criteria for participation in the study [46, 49, 50, 52–56, 60, 61, 63–67, 69, 72, 73, 83]. Twenty-five studies (54.3%) used a minimum age [40, 41, 43, 45, 46, 49, 50, 53, 54, 56, 58–60, 62, 64, 66, 67, 70, 72–74, 79–81, 83]. Eleven 11 studies (23.9%) used a maximum age in the inclusion and exclusion criteria [41, 43, 45, 46, 53, 54, 66, 67, 72, 74, 83]. The start date was not reported in 7 studies (15.2%) [39, 51, 57, 69, 73, 77, 78]. The end date was not reported in 6 studies (13%) [39, 51, 57, 69, 73, 77].

Participants with a concurrent diagnosis of MOH were included in 32 studies (69.6%) [38, 41, 43, 44, 46, 47, 49, 51–54, 56, 57, 59–63, 65–68, 71, 72, 74, 76, 77, 79, 81–83]. In those 32 studies, 13 declared the use of the ICHD-3 criteria for the diagnosis (40.6%) [40, 43, 44, 47, 51, 52, 57, 61, 65–68, 81].

Baseline period duration was 4 weeks for 11 studies (23.9%) [45, 50, 54, 55, 63, 69–71], 1 month in 9 studies (19.6%) [40, 47, 59, 62, 76, 77, 79–81], 3 months in 8 studies (17.4%) [51–53, 67, 72–74, 83] and 6 months in 1 study (2.2%) [64]. Seventeen studies (37.0%) did not mention the baseline period duration [38, 39, 42, 46, 48, 49, 56–58, 60, 61, 65, 66, 68, 75, 78, 82].

In 28 studies (60.9%), a minimum of 1 failed previous preventive drug was required to enter the study [38–41, 45, 47, 50, 54–56, 58–60, 62–66, 70–72, 74, 76–79, 82, 83]. Of those 28 studies, a minimum of 2 past previous preventive therapies was required in 8 studies (28.6%) [50, 54, 60, 65, 71, 79, 83], 3 past previous preventive therapies in 17 studies (60.7%) [38–41, 47, 56, 58, 59, 62–64, 66, 74, 76–78, 82] and 4 past previous preventive therapies in 3 studies (10.7%) [55, 70, 72].

A formal sample-size calculation was performed in 10 studies (21.7%) [40, 53, 57, 70, 78–83]. In 9 studies (19.6%) this was not done [38, 39, 41, 43, 47, 58, 59, 63, 76]. There was no information on sample size calculations in 27 reports (58.7%) [42, 44, 46, 48–52, 54–56, 60–62, 64–70, 72–75, 77].

### Treatment regimens

The following drugs and subcutaneous dosing schemes were used: erenumab 70 mg monthly (*n* = 23, 50%) [42, 45, 47–49, 51, 54, 56, 58–60, 62, 66–68, 70–73, 75, 77, 82, 83], galcanezumab 240 mg loading dose followed by 120 mg monthly (*n* = 21, 45.7%) [38–40, 47, 56, 58–61, 67, 69, 70, 74–76, 78–82], erenumab 140 mg monthly (*n* = 20, 43.5%) [38, 45, 47–49, 54, 56, 58–60, 62, 67, 69–72, 75–77, 82], erenumab 140 mg every 4 weeks (*n* = 11, 23.9%) [39, 43, 46, 50, 52, 53, 55–57, 63, 65], erenumab 70 mg every 4 weeks (*n* = 11, 23.9) [39, 41, 43, 46, 52, 53, 55–57, 64, 65], fremanezumab 225 mg monthly (*n* = 8, 17.4%) [44, 52, 56, 58, 60, 69, 70, 75] and fremanezumab 675 mg every three months (*n* = 3, 6.5%) [44, 56, 60]. There was a fixed starting dose for every participant in 31 studies (67%) [38, 41–44, 47, 50, 51, 53, 55, 58, 61–69, 71–74, 76, 78–81, 83], starting dose not fixed in 7 studies (15%) [46, 48, 49, 52, 54, 59, 60] and no information on this in 8 studies (17%) [39, 45, 56, 57, 70, 75, 77, 82]. Whether a dose increase was allowed was declared in 15 studies (32.6%) [41, 43, 46, 48, 53, 55, 58, 62, 65–69, 71, 72], with only 1 study utilizing a fixed dosing scheme [63]; 30 studies did not report if a dose increase was allowed [38–40, 42, 44, 45, 47, 49–52, 54, 56, 57, 59–61, 64, 70, 73–83]. Two studies reported that a dose decrease was allowed (4.3%) [41, 50], 1 study did not allow a dose decrease (2.2%) [62] and there was no information on this in the remaining 43 studies (93.5%) [38–40, 42–49, 51–61, 63–83]. The funding source for the drug treatment was mentioned in only 12 studies: public health care system (*n* = 6, 13%) [40, 50, 58, 59, 63, 83], pharmaceutical company (*n* = 4, 8.7%) [49, 53, 62, 76], patients themselves (*n* = 1, 2.2%) [77] and hospitals (*n* = 1, 2.2%) [79]; 34 studies (73.9%) did not mention the source of funding for the drugs [38, 39, 41–48, 51, 52, 54–57, 60, 61, 64–75, 78, 80–82].

Regarding concomitant migraine treatments, 21 studies allowed (45.7%) [41, 43, 44, 46, 47, 53, 58–62, 64, 66–68, 71, 72, 76, 77, 83] and 3 studies disallowed oral preventive medications (6.5%) [55, 69, 75]; no information on this was found in 22 studies (47.8%) [38–40, 42, 45, 48–52, 54, 56, 57, 63, 65, 70, 73, 74, 78–80, 82]. OnabotulinumtoxinA as concomitant therapy was allowed in 12 studies (26.1%) [41, 44, 46, 47, 58–60, 64, 72, 76, 77, 83] and disallowed in 3 studies (6.5%) [55, 61, 75]; no information on this was found in 30 studies (65.2%) [38–40, 42, 43, 45, 48–54, 56, 57, 62, 63, 65–71, 73, 74, 78–82]. In none of the studies information was found on concomitant use of transitional treatments or interventions (e.g. nerve blocks), neuromodulation or physical therapy.

There was no specific information on the management of MOH in 38 studies (82.6%) [38–46, 48, 50, 54–61, 64–66, 68–83], no intervention for MOH mentioned in 5 studies (15.6%) [47, 51–53, 63], education only in 1 study (3.1%) [49], education with inpatient withdrawal in 1 study (3.1%) [67] and education with outpatient withdrawal in 1 study (3.1%) [62].

### Definitions

Ten studies (21.7%) defined a migraine day for the study (Table 3) [39, 44, 45, 54, 55, 58, 62, 69, 70, 76], while only 5 studies defined a headache day for the study (10.9%) [39, 45, 55, 62, 76]. One study defined moderate-to-severe headache day [63]. No study defined a migraine attack or headache attack.

**Table 3:** Study definitions of a migraine day (*N* = 10)

Study	Definition
**Alpuente 2021 **[39]	“A migraine day was defined as any day with moderate–severe headache or/and headache with migraine features such as photophobia, phonophobia, nausea, and vomiting.”
**Barbanti 2022 **[44]	“A migraine day was defined as a calendar day characterized by > 4 consecutive hours of a migraine with or without aura or a headache of any duration successfully treated with migraine-specific acute medications (triptans).”
**Becker 2022 **[45]	“A migraine day was defined as any calendar day in which the participant experienced a qualified migraine headache (onset, continuation, or recurrence of the migraine headache). A qualified migraine headache was defined as: 1. A migraine headache, lasting for at least four continuous hours, and meeting criteria a and/or b: a) At least two of the following pain features: Unilateral, Throbbing, Moderate to severe, Exacerbated with exercise/physical activity b) At least one of the associated symptoms: • Nausea and/or vomiting • Photophobia and phonophobia OR 2. If the participant took a triptan or ergot-derivative on a calendar day, then it was considered as a migraine day regardless of the duration and pain features/associated symptoms.”
**De Matteis 2021 **[54]	“A ‘migraine day’ was defined accordingly to the ICHD-3 criteria.”
**De Vriesch Lentsch 2021 **[55]	“An automated and validated algorithm, based on the ICHD- 3 criteria, or intake of a triptan was used to determine whether headache days fulfilled migraine criteria.”
**Iannone 2022 **[58]	“A migraine day was defined as a calendar day with a headache meeting criteria for migraine (with or without aura) or a day when an acute migraine-specific medication (triptan or ergot) was used.”
**Lambru 2020 **[62]	“A ‘migraine day’ was defined according to the IHS classification criteria.”
**Raffaelli 2022 **[69]	“A migraine day was defined as any calendar day fulfilling the ICHD-3 criteria of a definite or probable migraine.”
**Raffaelli 2022 **[70]	“A migraine day was defined as any calendar day with a headache fulfilling the criteria of a definite or probable migraine according to the ICHD-3 classification.”
**Torres-Ferrus 2021 **[76]	“A migraine day was defined as any day with moderate–severe headache lasting at least 4 h or treated with analgesic. A headache day was defined as any headache lasting at least 30 min.”

### Headache diaries

Thirty-four studies (73.9%) mentioned the use of headache diaries [38–41, 43–45, 47, 49, 50, 52–55, 58–64, 66, 67, 69–72, 74, 76, 77, 79–83]. We found no information on headache diaries in 12 studies (26.1%) [42, 46, 48, 51, 56, 57, 65, 68, 73, 75, 77, 78]. A baseline headache diary was required in 31 studies (67.4%) [39–41, 43–45, 47, 49, 50, 53–55, 58, 59, 61–64, 66, 67, 69–72, 74, 76, 79–83]. Of the 34 studies describing the use of headache diaries, 6 reported using electronic headache diaries (17.6%) [38, 39, 45, 47, 55, 76], 7 reported paper diaries (20.6%) [41, 44, 49, 58, 72, 74] and 21 studies did not specify the modalities of the headache diaries (61.8%) [40, 43, 52–54, 59–64, 66, 67, 69–71, 79–83]. Twenty-seven of the 34 studies mentioned recording the use of acute medications in the diaries (79.4%) [39–41, 43–45, 47, 49, 50, 53–55, 58, 59, 61–63, 66, 67, 69, 70, 74, 76, 80–83].

### Baseline characteristics of participants

Age and sex were reported in all studies. Weight or BMI were reported in 12 studies (26.1%) [40–44, 57, 67, 79–82]; height only in 2 studies (4.3%) [73, 81]. Blood pressure was recorded at baseline in 2 studies only (4.3%) [62, 63]. Cardiovascular comorbidities were reported in 9 studies (19.6%) [41, 43, 48, 57, 67, 79–81, 83], gastro-intestinal comorbidities in 8 studies (17.4%) [40, 41, 43, 57, 67, 79–81] and psychiatric comorbidities in 18 studies [39–41, 43, 44, 53, 56, 57, 60, 61, 66–68, 76, 79–81, 83].

As for headache characteristics, 17 studies documented the age of headache onset (37.0%) [38, 40, 44, 45, 47–49, 53, 56, 61, 64, 67, 72, 74, 79, 80, 83]. Thirty studies reported the duration of CM (65.2%) [38–47, 49, 53, 54, 56, 58, 59, 61, 62, 65–68, 72–74, 76, 79–81, 83]. The presence of aura was reported in 15 studies (32.6%) [38, 39, 45–47, 49, 53, 58, 59, 62, 64, 69, 70, 76, 83], site of headache in 9 studies (19.6%) [39, 41, 43, 44, 47, 76, 79–81] and severity of pain in 19 studies (41.3%) [40–44, 47, 48, 54, 58, 59, 64–66, 74, 76, 79–81, 83]. Associated symptoms of headache were reported in 12 studies (26.1%) [40–44, 47, 48, 66, 76, 79–81], presence of premonitory symptoms in 3 studies (6.5%) [40, 41, 81] and presence of cranial autonomic symptoms in 7 studies (15.2%) [40, 41, 43, 44, 79–81].

Baseline information on response to onabotulinumtoxinA treatment for CM was reported in 14 studies (30.4%) [39, 41, 43, 44, 46, 47, 49, 57, 61–63, 66, 67, 80], and treatment response to triptans was documented in 10 studies (21.7%) [40–44, 47, 66, 79–81].

### Outcomes, endpoints and objectives

The most common primary endpoint/objective of the studies was change in monthly migraine days (*n* = 16, 34.8%) [38, 41–44, 46, 54, 55, 58, 62, 69–71, 78, 80, 82], followed by responder rate (*n* = 15, 32.6%) [38, 39, 45, 49, 50, 53, 54, 59, 61, 64–66, 72, 79, 81] and change in monthly headache days (*n* = 5, 10.9%) [38, 44, 60, 74, 80]. Other primary endpoints or objectives defined by the researchers were model building (*n* = 3, 6.5%) [47, 56, 83], change in acute medication intake (*n* = 3, 6.5%) [38, 54, 71], change in validated questionnaire or scale score (*n* = 3, 6.5%) [59, 75, 77], change in pain intensity (*n* = 2, 4.3%) [54, 74], conversion from CM to EM (*n* = 1, 2.2%) [40] and conversion from MOH to non-MOH (*n* = 1, 2.2%) [67]. Eight studies did not specifically define the primary endpoint/objective in the paper (17.4%) [48, 51, 52, 57, 63, 68, 73, 76]. Five studies declared multiple primary endpoints/objectives (10.9%) [38, 54, 59, 71, 74].

Migraine days were used by 37 studies (80.4%) as any endpoint [38–50, 52–59, 62–65, 68–70, 72, 75–78, 80–83]; 31 studies (67.4%) used headache days as any study endpoint [38, 39, 41, 43–50, 52–57, 60–64, 66, 67, 69–72, 76, 80]. A detailed overview can be found in Table 4.

**Table 4:** Headache-related endpoints

Headache-related endpoints	Number of studies present (%)
Migraine days	37 (80.4%) [38–50, 52–59, 62–65, 68–70, 72, 75–78, 80–83]
Acute treatment utilization	33 (71.7%) [38, 40–44, 47–49, 52–55, 57–59, 61, 62, 64–71, 77–83]
Headache days	31 (67.4%) [38, 39, 41, 43–50, 52–57, 60–64, 66, 67, 69–72, 74, 76, 80]
Intensity of headache	16 (34.8%) [38, 42–44, 52, 54, 57, 64, 68, 74, 76, 78–80, 82, 83]
Conversion of medication overuse to non-medication overuse	12 (26.1%) [40, 46, 47, 49, 51, 52, 60, 67, 72, 77, 79, 83]
Conversion to episodic migraine	10 (21.7%) [40, 41, 50, 62, 66, 74, 77, 79, 82, 83]
Moderate to severe headache days	4 (8.7%) [45, 61, 71, 79]
Crystal clear days	2 (4.3%) [61, 62]
Severe headache days	1 (2.2%) [63]

Thirty-nine studies (84.8%) presented responder rates to the drugs: 19 presented results on migraine days only (41.3%) [42, 46, 49, 50, 53–55, 58, 59, 62, 64, 65, 68, 70, 75, 78, 81–83], 9 on headache days only (19.6%) [52, 56, 60, 63, 66, 67, 71, 72, 74], 9 on both headache days and migraine days (19.6%) [38, 39, 41, 43–45, 47, 76, 80] and 2 on moderate to severe headache days (4.3%) [61, 79]. Fifteen studies used 1 percentage outcome only (14 studies with ≥ 50% responder rate [38, 39, 45–47, 49, 52, 53, 60, 65–67, 71, 82]; 1 study with ≥ 30% responder rate [75]), 4 studies with 2 percentage outcomes (≥ 50/75% responder rate in 3 studies [68, 74, 83]; ≥ 30/50% responder rate in 1 study [70]), 16 studies with 3 percentage outcomes (≥ 50/75/100% responder rate in 12 studies [41–44, 58, 59, 64, 76, 78–81]; ≥ 30/50/75 in 4 studies [50, 56, 63, 72]) and 4 studies with 4 percentage outcomes (≥ 30/50/75/100% responder rate in 5 studies [54, 55, 61, 62]).

No studies reported on onset of effect, cumulative hours per day of moderate to severe headache. One study (2.2%) evaluated the adherence to the treatment as one of the study’s endpoints [55]. No studies described pharmaco-economic endpoints.

Thirty-eight studies (82.6%) did not report using any anxiety/depression scales found in the list provided in the IHS guidelines. Two studies used 1 scale (4.3%) [53, 56], 4 used 2 scales (8.7%) [38, 47, 61, 76] and 2 studies used 3 scales (4.3%) [72, 74]. A detailed overview can be found in Table 5.

**Table 5:** Depression and anxiety scales

Depression and Anxiety	Number of studies present (%)
Beck Depression Inventory (BDI)	5 (10.9%) [38, 47, 72, 74, 76]
Beck Anxiety Inventory (BAI)	3 (6.5%) [38, 47, 76]
Generalized Anxiety Disorder (GAD-7)	1 (2.2%) [61]
Hospital Anxiety and Depression Scale (HADS)	2 (4.3%) [53, 56]
Hamilton Depression Rating Scale (HDRS)	2 (4.3%) [72, 74]
Hamilton Anxiety Rating Scale (HAM-A)	2 (4.3%) [72, 74]
Patient Health Questionnaire (PHQ-9)	1 (2.2%) [61]
State-train Anxiety Inventory (STA-I)	0

Nine studies (19.6%) did not report using a scale on patient’s reported outcome measures or healthcare outcomes/quality of life provided in the guideline by the IHS [39, 40, 50, 54, 60, 63, 64, 70, 71]. Seventeen studies used 1 scale (37%) [41–43, 45, 49, 51, 52, 55, 56, 62, 65, 66, 68, 69, 73, 77, 82], 13 used 2 scales (28.3%) [44, 46, 47, 57–59, 61, 67, 78–81, 83], 2 studies used 3 scales (4.3%) [48, 75], 4 studies used 4 scales (8.7%) [38, 53, 74, 76] and one study used 5 scales (2.2%) [72]. A detailed overview can be found in Table 6.

**Table 6:** Patient’s reported outcome measures and healthcare outcomes/quality of life

Patient’s reported outcome measures and healthcare outcomes/quality of life	Number of studies present (%)
Headache Impact Test (HIT-6)	32 (69.6%) [39, 41–44, 46–49, 53, 55–59, 61, 62, 65–69, 72, 74–76, 78–83]
Migraine Disability Assessment questionnaire (MIDAS)	22 (47.8%) [38, 44, 46–48, 51–53, 57–59, 61, 67, 72–74, 76, 78–81, 83]
Migraine-Specific Quality of Life questionnaire (MSQ v2.1)	7 (15.2%) [38, 45, 53, 72, 74, 76, 77]
Patient Global Impression of Change (PGIC)	6 (13%) [38, 45, 46, 55, 58, 76]
Allodynia Symptom Checklist (ASC-12)	4 (8.7%) [48, 53, 72, 74]
EuroQoL-5 Dimension Questionnaire (EQ-5D)	1 (2.2%) [75]
Short Form 12-Item Health Survey (SF12)	1 (2.2%) [75]
Functional Impairment Scale (FIS)	0
Migraine Functional Impact Questionnaire (MFIQ)	0

### Biomarker collection

No study collected saliva or cerebrospinal fluid for analysis. One study collected blood serum samples to determine polymorphic variants of calcitonin receptor-like receptor and receptor activity modifying protein 1 genes [83].

### Adverse events

Thirty-six studies reported adverse events (78.3%) [41, 43–53, 55, 58–68, 71–74, 76–81, 83]; 19 studies specifically reported on serious adverse events (41.3%) [41, 43, 45, 46, 50, 52, 62, 64–68, 71, 72, 74, 76–78, 83]. Twenty studies (43.5%) reported on reasons for discontinuation [41, 44, 46, 47, 49, 50, 55, 58, 61–65, 71, 72, 74, 77, 78, 80, 83]. Only 3 studies reported whether there were any pregnancies during treatment (6.5%) [49, 62, 76]. No study reported on ECG recording, neuroimaging or systematic blood pressure monitoring.

### Quality assessment

The results of the overall quality rating by applying the NHLBI Study Quality Assessment Tool for Before-After Studies are as follows (Table 7). Thirty-four studies were graded as “good” quality [38–41, 43–47, 50, 52–56, 58–61, 63–67, 70, 71, 74–76, 79–83]. Twelve studies were graded as “fair” [41, 48, 49, 51, 57, 62, 68, 69, 72, 73, 77, 78]. The details of the quality assessment are available as an online supplement.

**Table 7:** Quality rating by applying the NHLBI study quality assessment tool for before-after studies

Question	Yes	No	Cannot Determine/Not-recorded/Not-Available
1. Was the study question or objective clearly stated?	45	1	0
2. Were eligibility/selection criteria for the study population prespecified and clearly described?	44	2	0
3. Were the participants in the study representative of those who would be eligible for the test/service/intervention in the general or clinical population of interest?	46	0	0
4. Were all eligible participants that met the prespecified entry criteria enrolled?	14	13	19
5. Was the sample size sufficiently large to provide confidence in the findings?	10	5	31
6. Was the test/service/intervention clearly described and delivered consistently across the study population?	40	6	0
7. Were the outcome measures prespecified, clearly defined, valid, reliable, and assessed consistently across all study participants?	46	0	0
8. Were the people assessing the outcomes blinded to the participants' exposures/interventions?	0	46	0
9. Was the loss to follow-up after baseline 20% or less? Were those lost to follow-up accounted for in the analysis?	34	4	8
10. Did the statistical methods examine changes in outcome measures from before to after the intervention? Were statistical tests done that provided p values for the pre-to-post changes?	45	1	0
11. Were outcome measures of interest taken multiple times before the intervention and multiple times after the intervention (i.e., did they use an interrupted time-series design)?	34	11	1
12. If the intervention was conducted at a group level (e.g., a whole hospital, a community, etc.) did the statistical analysis take into account the use of individual-level data to determine effects at the group level?	0	1	45

## Discussion

To our knowledge this is the first systematic review in the field of migraine to analyse the methodology of RWE studies on preventive treatments. We have specifically focused on prospective, observational, clinic-based studies with anti-CGRP pathway monoclonal antibodies for the preventive treatment of both EM and CM.

The majority of studies were deemed to be of “good” or “fair” quality based on the quality assessment tool. These studies help the scientific community to create proper insights and inferences on the efficacy, tolerability and safety parameters of anti-CGRP pathway monoclonal antibodies use in real-world clinical settings. We did however find rather large heterogeneity on multiple methodological aspects such as endpoint determination, key definitions and longitudinal data recordings (e.g. the use of headache diaries). Interesting observations for future development of RWE studies in migraine will be discussed below.

Definitions are important aspects of clinical trials to help the interpretability, reproducibility of results and comparison between studies. Unfortunately our conclusion from this systematic analysis is that the large majority of RWE studies analysed do not provide the audience with definitions on migraine days and headache days. Only 10 studies (21.7%) defined a migraine day. After analysing the wordings most of the definitions rely on the ICHD-3 criteria for a migraine attack, since ICHD-3 does not contain formal criteria for a migraine day. Interestingly enough, definitions for migraine day and moderate/severe headache day are available in the clinical trial guidelines of the IHS but these were rarely used in the RWE studies [30, 31]. The lack of a formal definition of a migraine and/or a headache day in ICHD is all the more important as the change in monthly migraine days, the change in monthly headache days and the responder rate (which itself is related to migraine and/or headache days) are typical primary endpoints of this type of real-world studies. Our recommendation for a new iteration of ICHD would be to include a formal definition of migraine day (and perhaps headache day) based on consensus within the headache expert community; alternatively the IHS may develop a guideline for the conductance of RWE studies.

Headache diaries are indispensable for clinical research of headache treatments [30, 31]. Almost three-quarters of studies in our systematic review used them, but still a non-negligible number of studies are not mentioning their use. What is interesting is that 61.8% of studies using headache diaries did not mention the modalities of use and only 6 studies used electronic diaries. Digitization of society provides opportunities as digital headache diaries limit the amount of recall bias and provide more structured data on headache/migraine days and acute medication intake. Therefore, our results show there is room for improvement of RWE studies in terms of the quality of the recording of migraine/headache days.

The enrolment process in prospective observational studies is different from randomized-controlled trials. Most studies relied on consecutive enrolment of participants from headache clinics. A minority of studies (10 studies, 21.7%) performed a sample size calculation. However, sample size calculations in RWE studies are recommended to produce reliable results and to improve generalizability of the study, and data from phase 2 or phase 3 studies are available to that end.

Defining the primary endpoint is crucial for medical research, but this was missing in almost 1 in 5 studies. The majority of primary endpoints chosen are distributed across studies looking at reductions in migraine or headache days versus responder rates in terms of migraine and/or headache days (e.g. 30 or 50% reduction). Currently the headache community supports both types of endpoints [30, 31].

Surprisingly, very little attention was given to cardiovascular parameters (including blood pressure monitoring and ECG) in the studies. CGRP is a highly potent vasodilator and may act as a vasodilatory safeguard during cerebral and cardiac ischemia. Post-marketing retrospective analysis and a recent prospective follow-up study revealed signals of elevated blood pressure after exposure to certain anti-CGRP pathway monoclonal antibodies, a phenomenon that was not observed in the pivotal randomized clinical trials [85, 86]. As the aim of RWD is not only to assess effectiveness but also to provide safety information, we expected higher number of studies investigating cardiovascular safety. One reason may be that almost all of the included RWE studies in this systematic review were performed in Europe where the Summary of Product Characteristics (SmPC) of anti-CGRP pathway monoclonal antibodies does not require monitoring cardiovascular parameters, including blood pressure. While risk of hypertension is a matter of debate, it may be recommended for future RWE studies to include at least blood pressure monitoring.

Thirty-eight studies (82.6%) did not report using an anxiety/depression scale as part of the patients’ assessment, but on the contrary only nine studies (19.6%) did not report using a scale on patient’s reported outcome measures or healthcare outcomes/quality of life, as suggested in the guideline by the IHS. Our impression is that researchers aim to quantify the burden of disease from the migraine disorder but refrain to specifically look into the dimension of anxiety and depression. However anxiety and depression are highly prevalent comorbid disorders of migraine and bring additional burden to the patient. The inclusion of anxiety and depression scales helps the fine-grained analysis of the patient cohort but also gives way to additional insights into the treatment effect of the investigational products on these comorbid conditions. We should note that e.g. treatment with onabotulinumtoxinA leads to a significant reduction of both CM severity and comorbid major depressive disorder [87]. It is recommended to use validated scales for anxiety and depression in future RWE studies.

Strengths of this systematic review were the systematic approach by multiple investigators to use predefined protocol, research questions, entry forms and analysis methodology to tackle all tasks. Our analysis was limited to prospective, non-randomized observational studies so no inferences on other forms of RWE studies (e.g. retrospective studies or case series) can be made.

Two additional remarks should be made. First, the IHS, led by an international collective of clinical and scientific headache experts, has published guidelines for clinic-based headache registries. The document stipulates the importance and value of good quality clinic-based data for a wide variety of purposes which may serve many actors in healthcare in decision making steps. It stresses the importance of a formal research protocol to collect data in the best way possible. The experts acknowledge that RWD from well-designed headache registries can provide wide-ranging and novel insights into the characteristics, burden, and treatment of headache disorders and ultimately lead to improvements in the management of patients with headache [88]. We greatly appreciate the new guidelines and we hope our analysis encourages further efforts to improve rigorous designs of real-world studies in the field of headache disorders. Secondly, prospective, observational, clinic-based studies can be excellent settings for the exploration and testing of new outcome variables or study hypotheses. Therefore, by presenting our results, we do not insist on a single standard for all prospective, observational, clinic-based studies with formalized parameters but welcome new approaches, as long as they have been classified a priori as exploratory variables/outcomes and they have received ethical approval before the start of the study and data collection.

## Conclusion

This is the first systematic review on methodology of RWE studies in migraine, in particular regarding the preventive treatment with monoclonal antibodies against the CGRP pathway. We have identified multiple areas of potential improvement for future RWE studies, including the need for universal definitions of migraine/headaches, the use of (electronic) diaries, the calculation of sample sizes, and the use of anxiety and depression scales. In particular regarding CGRP pathway monoclonal antibodies systematic monitoring of blood pressure is recommended. We hope our analysis will be of benefit for future research, and ultimately patients with migraine.

## Supplementary Information

**Additional file 1.**

## Data Availability

The datasets used and/or analysed during the current study are available from the corresponding author on reasonable request.

## Abbreviations

CGRP: Calcitonin gene-related peptide
CM: Chronic migraine
EM: Episodic migraine
FDA: Food and Drug Administration (United States)
ICHD: International Classification of Headache Disorders
IHS: International Headache Society
MOH: Medication-overuse headache
RWD: Real-world data
RWE: Real-world evidence
SmPC: Summary of Product Characteristics
